# Cheerful Talk

**Published:** 1890-10

**Authors:** T. De Witt Talmage


### CHEERFUL TALK.

Have you ever realized what talk can do? This experiment has been made by medical scientists: A dozen men conspire to tell a well man he looks sick. They are to meet him on a journey, and by the time the fourth man is giving him melancholy salutation, he feels he is doomed; and the twelfth man comes up with his melancholy salutation just in time to help carry him home on a stretcher. Then twelve men conspire that they will meet a man in uncertain health, and tell him how well he looks. By the time the fourth man has met him with a cheerful salutation, his nervous system is all toned up, and by the time the twelfth man has met him with his cheerful salutation, he says to his wife: "Throw out that apothecary shop from our shelves; I don't want any more medicine." Now, the nation is only a man on a larger scale. If you want to prostrate business, and keep it prostrated, talk in a dolorous tone, and keep on talking. Let all the merchants sigh, and all the editors prognosticate hard times, and all the ministers groan in the pulpit. In the great orchestra of complaint, those who play the loudest trombones are those who have the fullest salaries and the completest wardrobe. They are only mad because they have to fall back upon the surplus resources of other years, or because they cannot make as large investments as they would like to make. Did you have your breakfast? Yes. Did you have your supper last night? Yes. Did you have a pillow to sleep on? Yes. What are you complaining about? The genuine sufferers, those who are really in destitution, for the most part suffer in silence; but the loudest cries against hard times are by the men to whom the times are not hard. Artists tell us it is almost impossible to sing well on a full stomach, but it has been demonstrated over and over again that it is possible for men to groan well on a full stomach. Stop singing Naomi and old Windham, and give us Mount Pisgah and Coronation.—*T. De Witt Talmage in the Observer.*

